# Supplementary material for: Sexual selection gradients change over time in a simultaneous hermaphrodite
Source: eLife. 2017 Jun 14;6:e25139. doi: 10.7554/eLife.25139 (PMC5511009; doi:10.7554/eLife.25139)
Supplement: Figure 2—source data 1. — DOI: http://dx.doi.org/10.7554/eLife.25139.005 [file elife-25139-fig2-data1.docx]

**Figure 2—source data 1.** The calculated values and their confidence interval (CI) for the opportunity for selection (*I*) and sexual selection (*I_s_*) for both sexual roles, indicated with subscript m or f, over the weeks.

| ***I*** | **Week** | **I-value** | **±CI** |
| --- | --- | --- | --- |
| *I_m_* | 1 | 2.64 | 0.0095 |
|  | 2 | 2.05 | 0.0033 |
|  | 3 | 1.11 | 0.0012 |
|  | 4 | 0.83 | 0.0007 |
|  | 5 | 0.66 | 0.0004 |
|  | 6 | 0.52 | 0.0003 |
|  | 7 | 0.42 | 0.0002 |
|  | 8 | 0.41 | 0.0002 |
| *I_f_* | 1 | 0.32 | 0.0011 |
|  | 2 | 0.30 | 0.0006 |
|  | 3 | 0.17 | 0.0003 |
|  | 4 | 0.12 | 0.0002 |
|  | 5 | 0.09 | 0.0001 |
|  | 6 | 0.09 | 0.0001 |
|  | 7 | 0.06 | 0.0001 |
|  | 8 | 0.07 | 0.0001 |
| *I_sm_* | 1 | 0.23 | 0.1346 |
|  | 2 | 0.10 | 0.0436 |
|  | 3 | 0.07 | 0.0249 |
|  | 4 | 0.05 | 0.0162 |
|  | 5 | 0.04 | 0.0120 |
|  | 6 | 0.04 | 0.0102 |
|  | 7 | 0.04 | 0.0081 |
|  | 8 | 0.03 | 0.0071 |
| *I_sf_* | 1 | 0.24 | 0.1244 |
|  | 2 | 0.17 | 0.0533 |
|  | 3 | 0.15 | 0.0354 |
|  | 4 | 0.15 | 0.0281 |
|  | 5 | 0.12 | 0.0208 |
|  | 6 | 0.10 | 0.0161 |
|  | 7 | 0.09 | 0.0127 |
|  | 8 | 0.05 | 0.0093 |
