# Supplementary material for: Sexual selection gradients change over time in a simultaneous hermaphrodite
Source: eLife. 2017 Jun 14;6:e25139. doi: 10.7554/eLife.25139 (PMC5511009; doi:10.7554/eLife.25139)
Supplement: Figure 2—source data 2. — For the top right of the table this shows the results for the regressions between relative male mating success (MSm) versus relative male reproductive success (RSm) and for bottom left (in italics) the results are shown for the regressions between relative principal component 2 (PC2) and relative male reproductive success (RSm). Each cell of the table comparing two slopes indicated the t-value, degrees of freedom (df) and significance (p). The first value column and row, respectively, indicate the sample size (N), the slope (β) and the standard error (SE) of the compared lines. The cells in the Week 1 row are left empty (-) because this line was not significant for relative MSm versus relative RSm and could thus not be compared with the other slopes. DOI: http://dx.doi.org/10.7554/eLife.25139.006 [file elife-25139-fig2-data2.docx]

**Figure 2—source data 2.** Slope comparisons between weeks for all the significant sexual selection gradients shown in Figures 2 and 3. For the top right of the table this shows the results for the regressions between relative male mating success (MS_m_) versus relative male reproductive success (RS_m_) and for bottom left (in italics) the results are shown for the regressions between relative principal component 2 (PC2) and relative male reproductive success (RS_m_). Each cell of the table comparing two slopes indicated the *t*-value, degrees of freedom (*df*) and significance (p). The first value column and row, respectively, indicate the sample size (*N*), the slope (*β*) and the standard error (*SE*) of the compared lines. The cells in the Week 1 row are left empty (-) because this line was not significant for relative MS_m_ versus relative RS_m_ and could thus not be compared with the other slopes.

|  |  | **relative MS_m_ versus relative RS_m_** | | | | | | | | |
| --- | --- | --- | --- | --- | --- | --- | --- | --- | --- | --- |
| ***Relative PC2 versus relative RS_m_*** | *N* |  | - | 24 | 23 | 23 | 23 | 22 | 22 | 17 |
|  | *β* |  | - | 1.78 | 2.71 | 2.67 | 3.03 | 2.40 | 2.38 | 2.26 |
|  | *SE* |  | - | 0.91 | 0.65 | 0.68 | 0.51 | 0.52 | 0.45 | 0.60 |
|  |  | **Week** | **1** | **2** | **3** | **4** | **5** | **6** | **7** | **8** |
|  | *25* | ***1*** | *t* | - | - | - | - | - | - | - |
|  | *1.87* |  | *df* | - | - | - | - | - | - | - |
|  | *0.76* |  | p | - | - | - | - | - | - | - |
|  | *24* | ***2*** | *0.28* |  | 0.83 | 0.04 | 0.60 | 0.59 | 0.41 | 0.44 |
|  | *2.21* |  | *45* |  | 43 | 42 | 42 | 42 | 41 | 37 |
|  | *0.95* |  | *0.78* |  | 0.41 | 0.97 | 0.55 | 0.56 | 0.69 | 0.66 |
|  | *23* | ***3*** | *0.69* | *0.32* |  | 0.04 | 0.39 | 0.38 | 0.41 | 0.51 |
|  | *2.60* |  | *44* | *43* |  | 43 | 42 | 41 | 41 | 36 |
|  | *0.73* |  | *0.49* | *0.75* |  | 0.68 | 0.70 | 0.71 | 0.69 | 0.61 |
|  | *23* | ***4*** | *0.66* | *0.31* | *0.01* |  | 0.42 | 0.32 | 0.35 | 0.46 |
|  | *2.61* |  | *44* | *43* | *42* |  | 42 | 41 | 41 | 36 |
|  | *0.83* |  | *0.51* | *0.75* | *0.99* |  | 0.68 | 0.75 | 0.73 | 0.65 |
|  | *23* | ***5*** | *0.95* | *0.54* | *0.25* | *0.22* |  | 0.87 | 0.94 | 0.98 |
|  | *2.85* |  | *44* | *43* | *42* | *42* |  | 41 | 41 | 36 |
|  | *0.69* |  | *0.35* | *0.59* | *0.80* | *0.83* |  | 0.39 | 0.35 | 0.33 |
|  | *22* | ***6*** | *0.49* | *0.10* | *0.30* | *0.29* | *0.60* |  | 0.02 | 0.18 |
|  | *2.33* |  | *43* | *42* | *41* | *41* | *41* |  | 40 | 35 |
|  | *0.54* |  | *0.63* | *0.92* | *0.77* | *0.77* | *0.55* |  | 0.99 | 0.86 |
|  | *22* | ***7*** | *0.53* | *0.12* | *0.29* | *0.28* | *0.60* | *0.03* |  | 0.17 |
|  | *2.35* |  | *43* | *42* | *41* | *41* | *41* | *40* |  | 35 |
|  | *0.46* |  | *0.60* | *0.90* | *0.77* | *0.78* | *0.55* | *0.98* |  | 0.87 |
|  | *17* | ***8*** | *0.07* | *0.34* | *0.75* | *0.72* | *1.01* | *0.56* | *0.61* |  |
|  | *1.79* |  | *38* | *37* | *36* | *36* | *36* | *35* | *35* |  |
|  | *0.78* |  | *0.94* | *0.74* | *0.46* | *0.48* | *0.32* | *0.58* | *0.55* |  |

N.B. The other comparisons between slopes were between Week 5 and 8 for relative PC1 and relative RS_m_ (*t* = 0.50, *df* = 37, *p* = 0.62) and Week 1 and 2 for relative PC2 and relative RS_f_ (*t* = 0.64, *df* = 45, *p* = 0.52). Please note that none of the comparisons reveal statistical differences between slopes.
