# Supplementary material for: Sexual selection gradients change over time in a simultaneous hermaphrodite
Source: eLife. 2017 Jun 14;6:e25139. doi: 10.7554/eLife.25139 (PMC5511009; doi:10.7554/eLife.25139)
Supplement: Figure 2—source data 3. — The values are only given for the significant (p < 0.05, black) and near-significant (p < 0.10, grey) slopes shown in Figures 2 and 3. DOI: http://dx.doi.org/10.7554/eLife.25139.007 [file elife-25139-fig2-data3.docx]

**Figure 2—source data 3.** Slope and confidence interval of the correlations between the different sexual selection measures. The values are only given for the significant (p < 0.05, black) and near-significant (p < 0.10, grey) slopes shown in Figures 2 and 3.

| **Beta** | **Week** | **Slope (β)** | **±CI** |
| --- | --- | --- | --- |
| *β_mm_* | 2 | 1.78 | 1.88 |
| *β_mm_* | 3 | 2.71 | 1.34 |
| *β_mm_* | 4 | 2.67 | 1.41 |
| *β_mm_* | 5 | 3.03 | 1.06 |
| *β_mm_* | 6 | 2.40 | 1.08 |
| *β_mm_* | 7 | 2.38 | 0.94 |
| *β_mm_* | 8 | 2.26 | 1.27 |
| *β_fm_* | 1 | 0.40 | 0.47 |
| *β_fm_* | 2 | 0.80 | 0.68 |
| *β_mf_* | 1 | 1.41 | 1.28 |
| *β_ff_* | 6 | -0.37 | 0.40 |
| *β_ff_* | 7 | -0.34 | 0.37 |
| *β_ff_* | 8 | -0.52 | 0.58 |
|  |  |  |  |
| *β_mPC1_* | 5 | -0.95 | 0.92 |
| *β_mPC1_* | 8 | -1.22 | 1.09 |
| *β_fPC1_* | 6 | -0.38 | 0.40 |
| *β_fPC1_* | 7 | -0.35 | 0.37 |
| *β_fPC1_* | 8 | -0.51 | 0.56 |
| *β_mPC2_* | 1 | 1.87 | 1.57 |
| *β_mPC2_* | 2 | 2.21 | 1.98 |
| *β_mPC2_* | 3 | 2.60 | 1.51 |
| *β_mPC2_* | 4 | 2.61 | 1.72 |
| *β_mPC2_* | 5 | 2.85 | 1.43 |
| *β_mPC2_* | 6 | 2.33 | 1.13 |
| *β_mPC2_* | 7 | 2.35 | 0.97 |
| *β_mPC2_* | 8 | 1.79 | 1.67 |
| *β_fPC2_* | 1 | 0.73 | 0.52 |
| *β_fPC2_* | 2 | 1.01 | 0.71 |
