# Supplementary material for: Sexual selection gradients change over time in a simultaneous hermaphrodite
Source: eLife. 2017 Jun 14;6:e25139. doi: 10.7554/eLife.25139 (PMC5511009; doi:10.7554/eLife.25139)
Supplement: Figure 3—source data 1. — PC1 represents the sexual bias (the relative difference between MSm and MSf); PC2 represents the overall mating activity (the correlation component between MSm and MSf). DOI: http://dx.doi.org/10.7554/eLife.25139.010 [file elife-25139-fig3-data1.docx]

**Figure 3—source data 1.** Results of the principal component analysis (PCA) for each week of the experiment. PC1 represents the sexual bias (the relative difference between MS_m_ and MS_f_); PC2 represents the overall mating activity (the correlation component between MS_m_ and MS_f_).

|  | **PCA** | **Eigenvalue** | **Percentage** | **Eigenvector MS_m_** | **Eigenvector MS_f_** |
| --- | --- | --- | --- | --- | --- |
| Week 1 | PC1 | 0.319 | 66.9 | -0.682 | 0.732 |
|  | PC2 | 0.158 | 33.1 | 0.732 | 0.682 |
| Week 2 | PC1 | 0.187 | 69.0 | -0.377 | 0.926 |
|  | PC2 | 0.084 | 31.0 | 0.926 | 0.377 |
| Week 3 | PC1 | 0.157 | 71.6 | -0.266 | 0.964 |
|  | PC2 | 0.062 | 28.4 | 0.964 | 0.266 |
| Week 4 | PC1 | 0.158 | 80.5 | -0.288 | 0.957 |
|  | PC2 | 0.038 | 19.5 | 0.957 | 0.288 |
| Week 5 | PC1 | 0.124 | 78.3 | -0.302 | 0.953 |
|  | PC2 | 0.034 | 21.7 | 0.953 | 0.302 |
| Week 6 | PC1 | 0.102 | 71.3 | -0.122 | 0.993 |
|  | PC2 | 0.041 | 28.7 | 0.993 | 0.122 |
| Week 7 | PC1 | 0.088 | 71.1 | -0.047 | 0.999 |
|  | PC2 | 0.036 | 28.9 | 0.999 | 0.047 |
| Week 8 | PC1 | 0.059 | 71.3 | -0.364 | 0.831 |
|  | PC2 | 0.024 | 28.7 | 0.831 | 0.364 |
